# Wound exudate reduction from retroperitoneum with facilitation of healing by triamcinolone injection: A case report

**DOI:** 10.1097/MD.0000000000031464

**Published:** 2022-11-04

**Authors:** Kazuhiro Tsunekawa, Shunsuke Yuzuriha

**Affiliations:** School of Medicine, Shinshu University, Nagano, Japan.

**Keywords:** exudate, moist environment, retroperitoneum, secondary healing, skin ulcer triamcinolone

## Abstract

Maintaining a moderately moist environment is important in the treatment of chronic skin ulcers. Excessive exudate inhibits wound healing, although it may be difficult to control. We report a case of successful treatment with triamcinolone to suppress retroperitoneal exudates and facilitate wound healing.

**Patient concerns:**

A 78-year-old man presented to our department with excessive exudative oozing from a chronic ulcer in the gluteal region. The retroperitoneum was exposed for a prolonged period after colon cancer surgery and developed chronic granulation.

**Diagnoses:**

The exposed retroperitoneum was covered using an island flap, with the left superior gluteal artery perforator providing the feeding vessels and skin graft, which covered part of the flap harvesting area. The flap and lower bed did not adhere because of the presence of an excessive retroperitoneal exudate. Skin grafts to the flap harvesting area were not accepted, and secondary healing did not proceed because of exudate leakage.

**Intervention:**

A single dose of 2.5-mg triamcinolone was injected inferiorly to the flap.

**Outcomes:**

After injection of triamcinolone, the exudate decreased, and secondary healing in the residual ulcer progressed satisfactorily.

**Conclusion:**

Injection of triamcinolone might be an option for controlling exudates to heal skin ulcers.

## 1. Introduction

Adequate moisture in the environment is an important factor for wound healing.^[1]^ Various ointments and wound dressings are used to maintain sufficient moisture in and around the wound according to the amount of wound exudate present. However, excessive and uncontrolled exudates may occasionally inhibit wound healing.

There are several causes of a large amount of exudate, including inflammation, damage to the lymphatic vessels, and hypoalbuminemia.^[2,3]^ Studies have reported the use of triamcinolone to control excessive exudates due to certain causes. Local injection of triamcinolone has been reported to reduce pericardial fluid, ascites, and subcutaneous seroma formation after latissimus dorsi flap harvesting and abdominoplasty.^[4–8]^

This report describes a case in which triamcinolone was injected locally, effectively decreasing exudate from the retroperitoneum that had been exposed for a prolonged period, thereby leading to wound closure.

## 2. Case presentation

A 78-year-old man visited our hospital for management of surgical wound dehiscence. Miles surgery was performed for colon cancer (T3N0M0, stageIIA) at another hospital five years before the patient’s first visit to the hospital. Chemotherapy and radiation therapy were performed for lymph node metastasis one year and six months earlier, respectively, and resection of the tumor with the sacrum was performed six months earlier. The surgical wound did not heal and the retroperitoneum was exposed.

**Figure 1:**
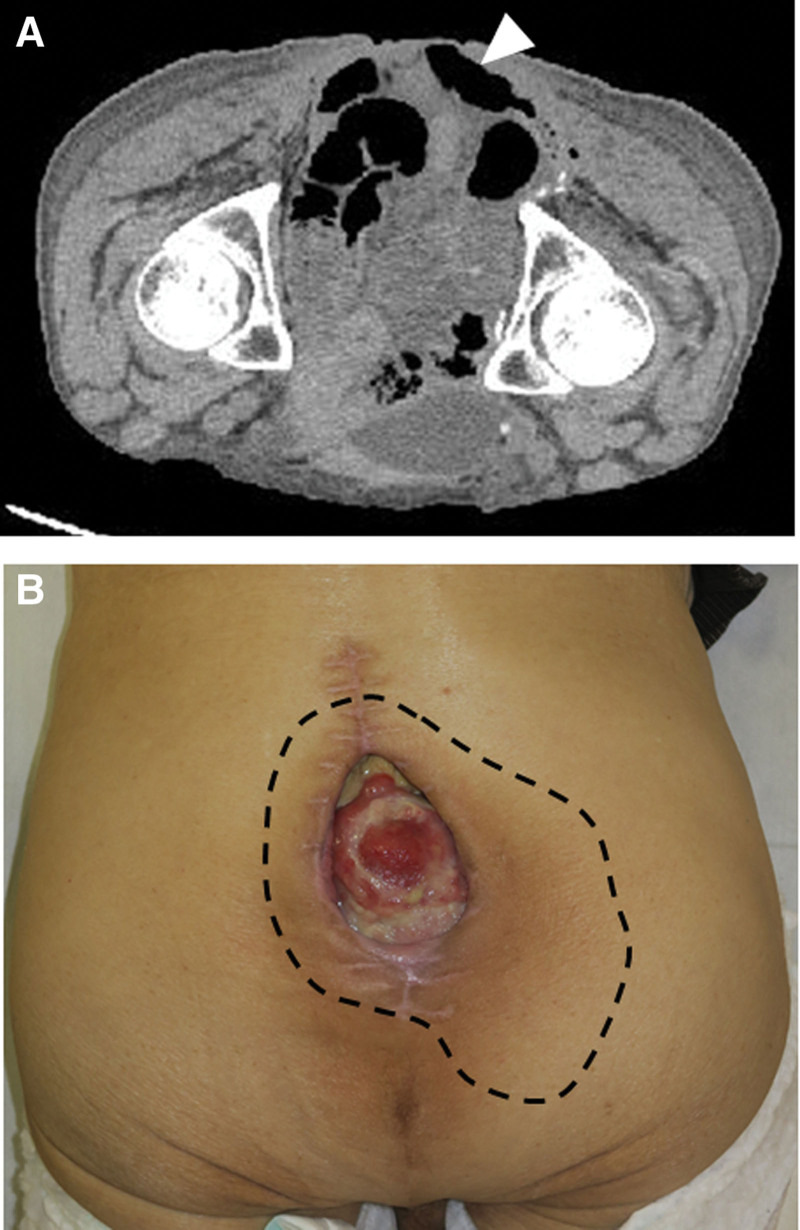
(a) Patient’s wound at first visit to outpatient clinic of plastic surgery department. After resection of the rectus and part of the pelvis, the surgical scar showed dehiscence and the retroperitoneum was exposed. The size of the ulcer was 5.5 × 4.5 cm and it had a pocket width of 16 × 12 cm (area surrounded by the dotted line); (b) Computed tomography image of the wound recorded with patient in the prone position, prior to surgery. The retroperitoneum is exposed (outside the body), and the intestinal tract is located directly below the wound (arrowhead).

At the first visit to the plastic surgery department, a 5.5 × 4.5 cm area of the retroperitoneum was exposed at the midline of the gluteal region, and a subcutaneous pocket measuring 16 × 12 cm was found around the exposed retroperitoneum. Infected granulation with a large amount of exudate was observed on the exposed retroperitoneum (Fig. 1a). Computed tomography revealed adhesions between the retroperitoneum exposed on the body surface and the intestinal tract (Fig. 1b). A gastrointestinal surgeon advised that debridement to freshen the granulation tissue on the retroperitoneum carries a considerable risk of intestinal damage that would be difficult to repair.

**Figure 2:**
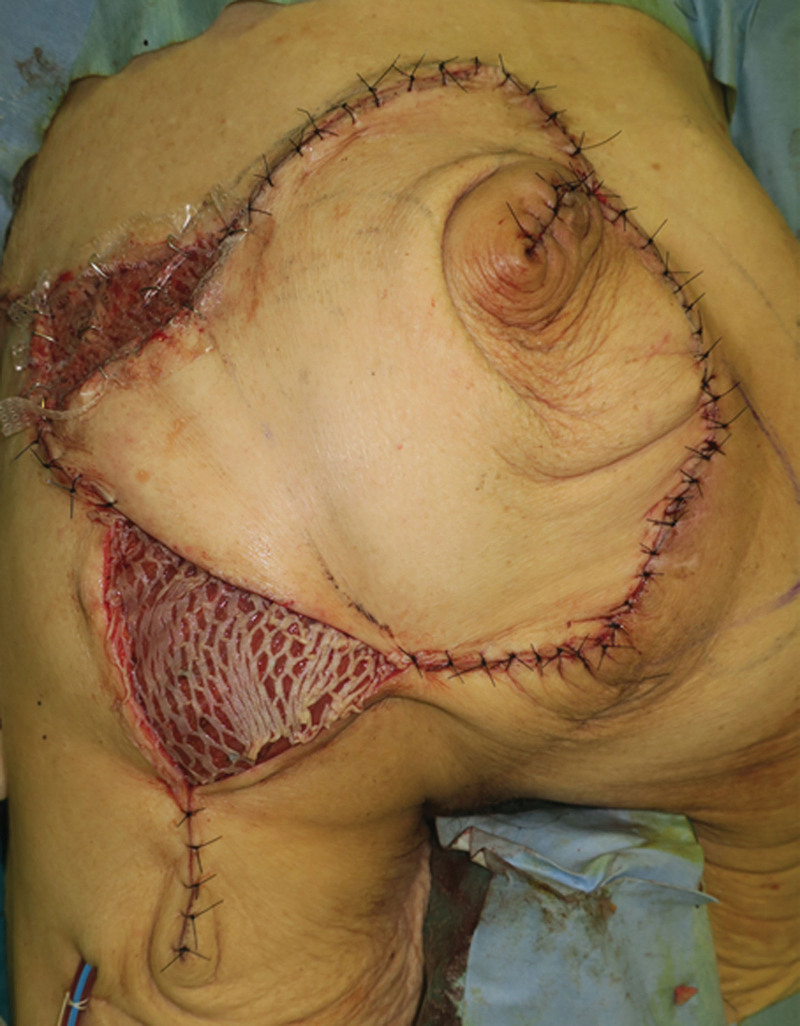
Island flap with left superior gluteal artery perforator and skin flap surgery. An island flap from the left buttock covered the peritoneum and filled the subcutaneous dead space. The mesh skin graft covers the cranial and caudal sides and cannot be closed using primary closure.

**Figure 3:**
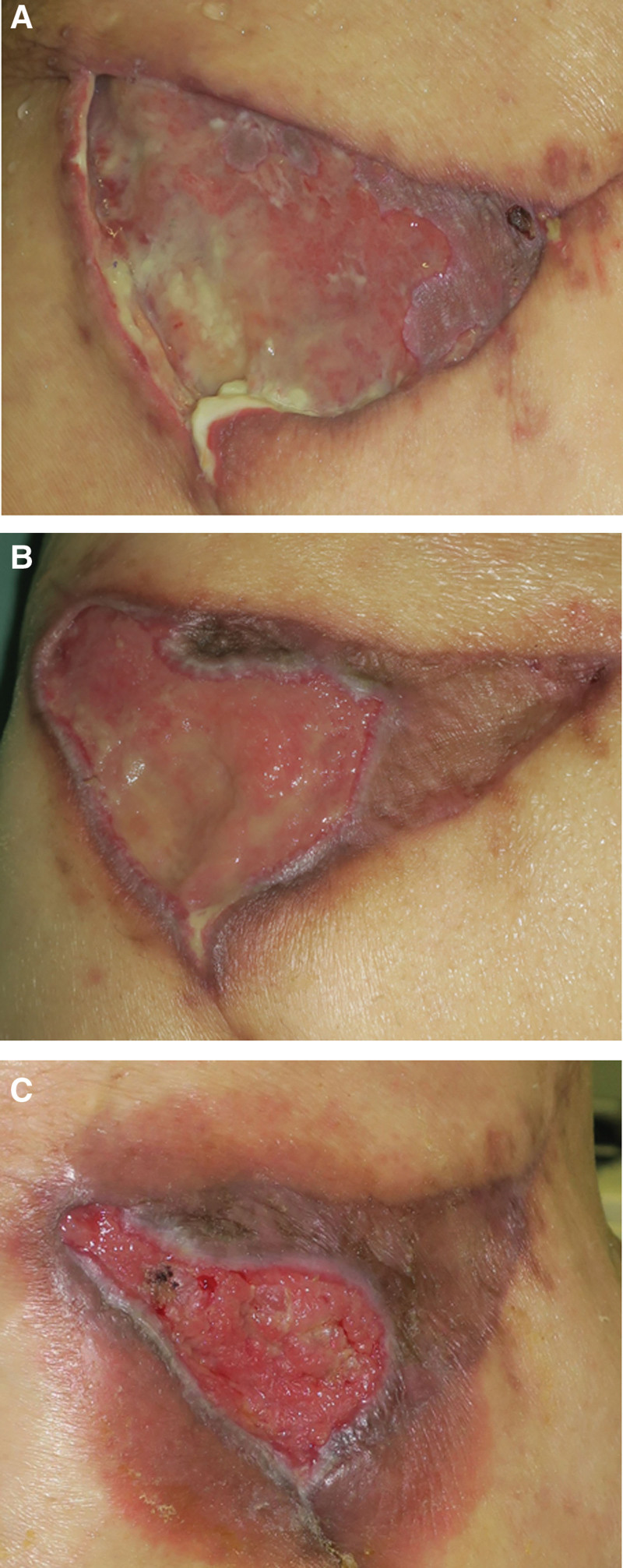
Skin graft on the flap harvesting area through the course of treatment. (a) Post flap surgery day 25: the skin graft on the cranial side barely survived. Much slough was present, and the wound surface was macerated due to exudation. (b) Post flap surgery day 69: the epithelium was slightly extended, but the slough remained, and the wound surface was macerated. Triamcinolone was injected on the same day. (c) Post flap surgery day 86 (17 days after injecting triamcinolone): the slough did not cover the wound surface. Good granulation formed and secondary healing progressed.

Surgery for wound closure without freshening the granulation tissue on the retroperitoneum was planned. An island flap with the left superior gluteal artery perforator as the feeding vessel was used to cover the dehiscence, and the dead space was filled using a de-epithelized flap (Fig. 2). Meshed split-thickness skin grafting was performed at the donor site that could not be reefed. The blood flow to the flap was good, but a large amount of exudate was identified between the flap and lower bed of the wound. The skin graft failed because of excessive exudate, and 77% of the flap donor site, which could not be reefed, remained as a skin ulcer (post-flap surgery day 25, Fig. 3a). After conservative treatment with an ointment made of water-absorbent base material and negative pressure wound therapy for five weeks, a second skin graft was performed, which also failed. Subsequently, fluid accumulation under the flap was punctured, and 70 mL of fluid was aspirated.

A single dose of 2.5-mg triamcinolone was injected at the non-united gap between the flap and lower bed using the outer cylinder of a blood vessel indwelling needle on post-flap surgery day 69 (Fig. 3b). At this stage, 64% of skin ulcers were retained.

**Figure 4:**
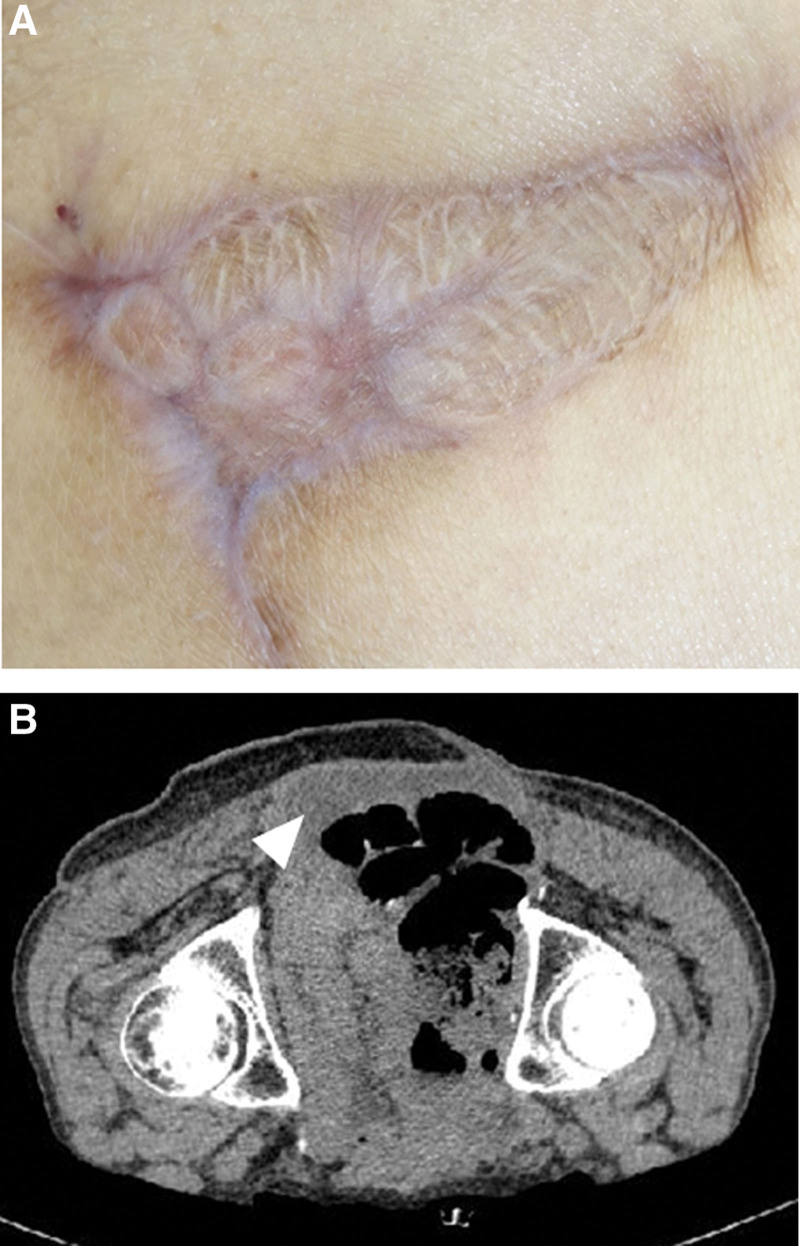
(a) Post flap surgery day 307. Secondary healing was complete; the skin ulcer did not recur; (b) Computed tomography image of wound site at post flap surgery day 307 with patient in the prone position. A small amount of fluid retention is observed between the retroperitoneum and flap (arrowhead). The retroperitoneum is not exposed to the external environment.

From the day after triamcinolone administration, wound exudate decreased markedly. On days 86 and 111 post-flap surgery, the ulcer had shrunk to 39% (Fig. 3c) and 14% of its original size, respectively. On postoperative day 146, the ulcer had closed. The ulcer did not recur; 10 months after the flap surgery, computed tomography showed a small amount of serous fluid retention (Fig. 4a and b).

The patient provided written informed consent to publish the details of the case and use the associated images.

## 3. Discussion

Triamcinolone injection to reduce the amount of exudate from the retroperitoneum accelerated the secondary wound contraction in this case. Chronic granulation of the patient’s peritoneum was exposed for six months. A large amount of exudate is also observed. Exudates from chronic ulcers contain degraded vitronectin and fibronectin, which inhibit adhesion between cells and diffusion of fibroblasts^[9]^; and may also contain fewer growth-promoting factors (platelet-derived growth factor, interleukin-6, tumor growth factor (TGF)-α, and TGF-β) than exudates from acute ulcers.^[1]^ Thus, the exudate from the chronic ulcer in this case contained several elements that could inhibit epithelialization and cell elongation.

These are the key factors for healing chronic ulcers, and wound bed preparation can be achieved by debridement, bacterial control, and exudate control.^[1]^ Debridement of chronic granulation on the peritoneum in this case was initially planned but could not be performed because of the risk of damage to the intestinal tract beneath the wound surface. This may be one reason why excessive exudation could not be reduced, preventing adhesion between the flap and lower bed.

Topical administration of triamcinolone has been proposed as an effective treatment for seroma after latissimus dorsi flap harvesting and abdominoplasty.^[7,8]^ Triamcinolone may prevent seroma from oozing into the dead space due to decreased vascular permeability and has an anti-inflammatory action that suppresses arachidonic acid synthesis, which plays a key role in the production of prostaglandins and leukotrienes.^[10]^ In the case presented here, the exudate from the chronic granulation might have immersed the skin wound due to flap harvesting because of the absence of adhesion between the flap and peritoneum. The excessively moist environment of the skin defect due to exudate from the retroperitoneum improved after injecting the patient with triamcinolone. Secondary wound healing may have been promoted by the formation of a suitably moist environment and fewer inhibiting factors for wound healing.

Triamcinolone has side effects that may cause infection; triamcinolone injected through a needle that initiates a connection between the peritoneum and island flap may cause peritonitis or intra-abdominal infections. No complications occurred owing to the use of triamcinolone. The appropriate dosage of triamcinolone injection around the peritoneum to reduce exudation cannot be proposed based on this solitary case and is a topic for future research.

In this case, triamcinolone was injected using only the outer cylinder of the indwelling needle, which was inserted in the gap between the flap and peritoneum. Attention should be paid to avoid peritoneal and intestinal tract damage when injecting triamcinolone with a needle in similar cases. Echographic guidance to administer the injection may be more conservative.

Although a moderately moist environment is a principal factor in treating chronic ulcers, the authors injected triamcinolone to solve the problem of local hyperexudation. Secondary healing was achieved in this case; however, further studies are needed to determine the complications, dose, and indications for the use of this agent. The use of triamcinolone to reduce serous fluid between the retroperitoneum and the subcutaneous tissue remains controversial.

## 4. Conclusion

The authors present a case in which local injection of triamcinolone was effective in controlling exudate from the retroperitoneum. Consequently, wound closure was achieved. The appropriate dosage of triamcinolone for injection is a topic for further study.

## Acknowledgments

The authors thank the participants for their involvement in the study.

## Author contributions

**Supervision:** Shunsuke Yuzuriha.

**Writing – original draft:** Kazuhiro Tsunekawa.

**Writing – review &amp; editing:** Kazuhiro Tsunekawa.
